# Predictors of positive airway pressure therapy termination in the first year: analysis of big data from a German homecare provider

**DOI:** 10.1186/s12890-018-0748-8

**Published:** 2018-12-05

**Authors:** Holger Woehrle, Michael Arzt, Andrea Graml, Ingo Fietze, Peter Young, Helmut Teschler, Joachim H. Ficker

**Affiliations:** 1 Sleep and Ventilation Center Blaubeuren, Respiratory Center Ulm, Ulm, Germany; 2 Department of Internal Medicine II, University Hospital Regensburg, Regensburg, Germany; 3 ResMed Science Center, ResMed Germany, Martinsried, Germany; 4 Charité – University Medical Center Berlin, Center for Cardiovascular and Vascular Medicine, Interdisciplinary Sleep Medicine Center, Berlin, Germany; 5 Clinic for Sleep Medicine and Neuromuscular Diseases, University Hospital Münster, Münster, Germany; 6 Department of Pneumology, Ruhrlandklinik, West German Lung Center, University Hospital Essen, University Duisburg-Essen, Essen, Germany; 7 Department of Respiratory Medicine, Allergology and Sleep Medicine, General Hospital Nuremberg, Nuremberg, Germany; 8 Paracelsus Medical University, Nuremberg, Germany

**Keywords:** Positive airway pressure, Compliance, Patient phenotype, Therapy termination

## Abstract

**Background:**

There is a lack of robust data about factors predicting continuation (or termination) of positive airway pressure therapy (PAP) for sleep apnea. This analysis of big data from a German homecare provider describes patients treated with PAP, analyzes the therapy termination rate over the first year, and investigates predictive factors for therapy termination.

**Methods:**

Data from a German homecare service provider were analyzed retrospectively. Patients who had started their first PAP therapy between September 2009 and April 2014 were eligible. Patient demographics, therapy start date, and the date of and reason for therapy termination were obtained. At 1 year, patients were classified as having compliance-related therapy termination or remaining on therapy. These groups were compared, and significant predictors of therapy termination determined.

**Results:**

Of 98,329 patients included in the analysis, 11,702 (12%) terminated PAP therapy within the first year (after mean 171 ± 91 days). There was a U-shaped relationship between therapy termination and age; therapy termination was higher in the youngest (< 30 years, 15.5%) and oldest (≥ 80 years, 19.8%) patients, and lower in those aged 50–59 years (9.9%). Therapy termination was significantly more likely in females versus males (hazard ratio 1.48, 95% confidence interval 1.42–1.54), in those with public versus private insurance (1.75, 1.64–1.86) and in patients whose first device was automatically adjusting or fixed-level continuous positive airway pressure versus bilevel or adaptive servo-ventilation (1.28, 1.2–1.38).

**Conclusions:**

This analysis of the largest dataset investigating PAP therapy termination identified a number of predictive factors. These can help health care providers chose the most appropriate PAP modality, identify specific patient phenotypes at higher risk of stopping PAP and target interventions to support ongoing therapy to these groups, as well as allow them to develop a risk stratification tool.

## Background

Poor compliance with long-term therapies compromises the effectiveness of treatment and, on average, half of patients with a chronic illness don’t adhere to their prescribed therapy [1]. Continuous positive airway pressure (CPAP) is the gold standard treatment for obstructive sleep apnea (OSA). However, long-term compliance with CPAP therapy is important for the achievement of therapeutic goals, including improvements in daytime sleepiness [2–6] and memory [7], reductions in blood pressure and the incidence of hypertension [8–13], and decreased cardiovascular risk [9, 14, 15]. The Sleep Apnea cardioVascular Endpoints (SAVE) study (NCT00738179) was the first large randomized controlled trial to investigate the effects of CPAP therapy on morbidity and mortality in patients with sleep apnea at risk for cardiovascular events [16]. The trial included nonsleepy patients with OSA who were randomized to CPAP or usual care. The results showed no significant difference in the rates of hospitalization or mortality between the CPAP and usual care groups. However, mean CPAP usage was only 3.3 h/night during the trial, and this low compliance might have contributed to the negative result of this study.

Rates of noncompliance with CPAP (defined as device use for < 4 h/night) have been reported to range from 29 to 83% in OSA patients receiving long-term therapy [17]. Although a number of studies have investigated compliance with positive airway pressure (PAP) therapy, the results have not always been consistent [18–24]. In addition, while the pattern of compliance within first weeks of CPAP therapy appears to be predictive of longer term compliance [25] – highlighting the importance of achieving good compliance early to ensure adequate long-term device use – there is a general lack of robust data from big data analyses [26] on specific predictors of PAP therapy persistence or termination. Identifying patients who are at risk of stopping CPAP therapy and the time course of when this might occur could help to optimize and target the provision of support strategies designed to increasing compliance [17, 19, 27, 28].

Therapy termination, with return of the PAP device to the service provider, represents a definitive form of noncompliance. Even though noncompliant, a patient who retains the device still has the potential to re-start therapy. This is much less likely once the device has been returned. However, very little data exist on the rates of therapy termination in patients using PAP therapy.

This big data analysis uses information from the database of a German homecare provider to describe the patient population treated with PAP therapy, analyze termination rates over the first year of therapy, and investigate factors predictive of therapy termination.

## Methods

### Patient population/sample

Observational study data were obtained from the database of a Germany homecare service provider (ResMed Healthcare Germany). Patients who had a physician diagnosis of sleep apnea and were prescribed PAP therapy, started PAP therapy for the first time between 1 September 2009 and 30 April 2014, and were being treated with fixed-pressure CPAP, automatically adjusting continuous positive airway pressure (APAP), bilevel PAP or adaptive servo-ventilation (ASV) devices, using a nasal mask, nasal pillows or a full face mask interface, were eligible for inclusion in this analysis. The presence of sleep apnea was based on diagnosis by each patient’s treating physician.

### Data extraction and definitions

The commercial homecare provider database contains information relevant to the provision of PAP therapy rather than individual clinical data. Therefore, it stores less information than a full electronic medical record, and did not record the severity of sleep apnea, mode of diagnosis, and comorbidities. The following variables were extracted from the database for each patient: therapy start date, and the date of and reason for therapy termination. A de-identified copy of all information available (to protect patient privacy) was provided to the scientific committee analyzing the data. German data protection law allows for the use of such data, if strictly anonymized, for scientific purposes. Therefore, patient informed consent and ethical approval were not required.

Therapy termination was then described as compliance related if it occurred as the result of patient decision or behavior due to patient-reported problems with the PAP interface or device (i.e. not accepting or tolerating PAP therapy). Terminations that occurred when a patient was lost to follow-up, transferred to a ventilation device or died, or were related to insurance coverage issues or patient transfer to another homecare provider were classified as not compliance related.

**Fig. 1:**
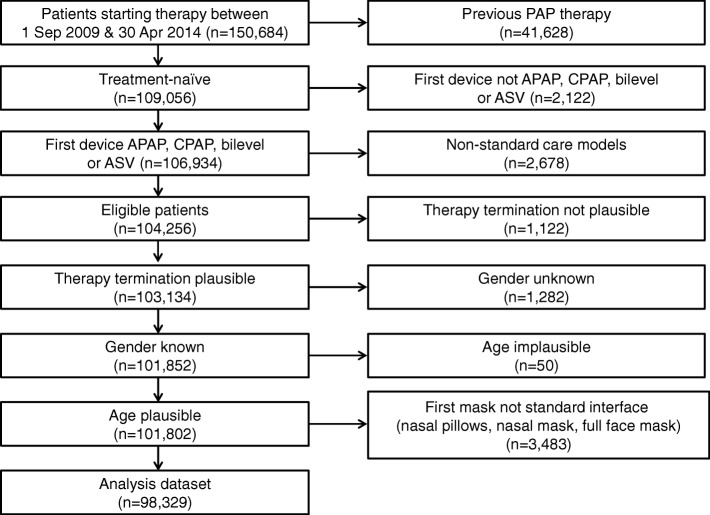
Flow diagram of patient selection

Patients were assigned to one of two groups based on their PAP usage status at 1 year: on therapy or therapy terminated. Data were censored whereby all patients who had not terminated therapy by 1 year were assigned to the “on therapy” group. All included patients had data available on the status at last observation (event occurrence or censoring) and time to event (or censoring). A flow diagram showing the patient selection pathway is presented in Fig. 1.

### Data analysis

Age was regarded as plausible if the value was between 0 and 100 years, and termination date was regarded as plausible if it took place between therapy start date and the date that data were extracted. Several variables were used as covariates in the model (i.e. gender, age, insurance, and device), and complete observations were required for these data.

Numerical data are presented as mean ± standard deviation (SD). Differences between groups were analyzed using a t-test because the patient numbers allow for normal approximation. Ordinal and nominal data was presented as absolute and relative frequency. Differences in proportion were assessed with Z-tests. The time-to-event data were analyzed using Kaplan-Meier-plots and Cox proportional hazards regression. An event was defined as the compliance-related termination of PAP therapy, whereas compliance not related to therapy termination was defined as right censoring. Time was defined as time on therapy in the first year (i.e. time between therapy start registered in the ResMed database system and time of therapy termination, or 365 days for patients who did not terminate therapy). For compliance-related therapy termination, this is time to termination and for compliance not related to termination, this is time to censoring.

In general, *p* values of < 0.05 were considered statistically significant. All statistical analyses were performed using IBM SPSS Statistics 22 and R version 2.15.

## Results

A total of 98,329 patients were included in the analysis dataset (Fig. 1). Of these, 12% (*n* = 11,702) terminated PAP therapy within the first year. Mean time to therapy termination was 171 ± 91 days. Available demographic and clinical data for patients who continued PAP therapy or had compliance-related termination within the first year are shown in Table 1. Patients who terminated therapy were significantly older, significantly more likely to be female and to have APAP or CPAP as their first PAP device, and significantly less likely to have private insurance compared with those who remained on therapy (Table 1).

**Table 1:** Baseline demographic and clinical characteristics of patients who continued or terminated positive airway pressure therapy in the first year

	On Therapy (*n* = 86,627)	Terminating therapy (n = 11,702)	Total (*n* = 98,329)
Age, years	61 ± 12	63 ± 13*	61 ± 13
Gender, n (%)			
Male	66,755 (77)	8063 (69)*	74,818 (76)
Female	19,872 (23)	3639 (31)*	23,511 (24)
Insurance, n (%)			
Public	73,949 (85)	10,694 (91)*	84,643 (86)
Private	12,678 (15)	1008 (9)*	13,686 (14)
First PAP device, n (%)			
APAP/CPAP	78,357 (90)	10,827 (93)*	89,184 (91)
Bilevel/ASV	8270 (10)	875 (7)*	9145 (9)

Values are mean ± standard deviation, or number of patients (%)

*p < 0.05 vs patients who remained on therapy

*APAP* automatic continuous positive airway pressure, *ASV* adaptive servo-ventilation, *Bilevel* bilevel positive airway pressure, *CPAP* continuous positive airway pressure, *PAP* positive airway pressure

**Fig. 2:**
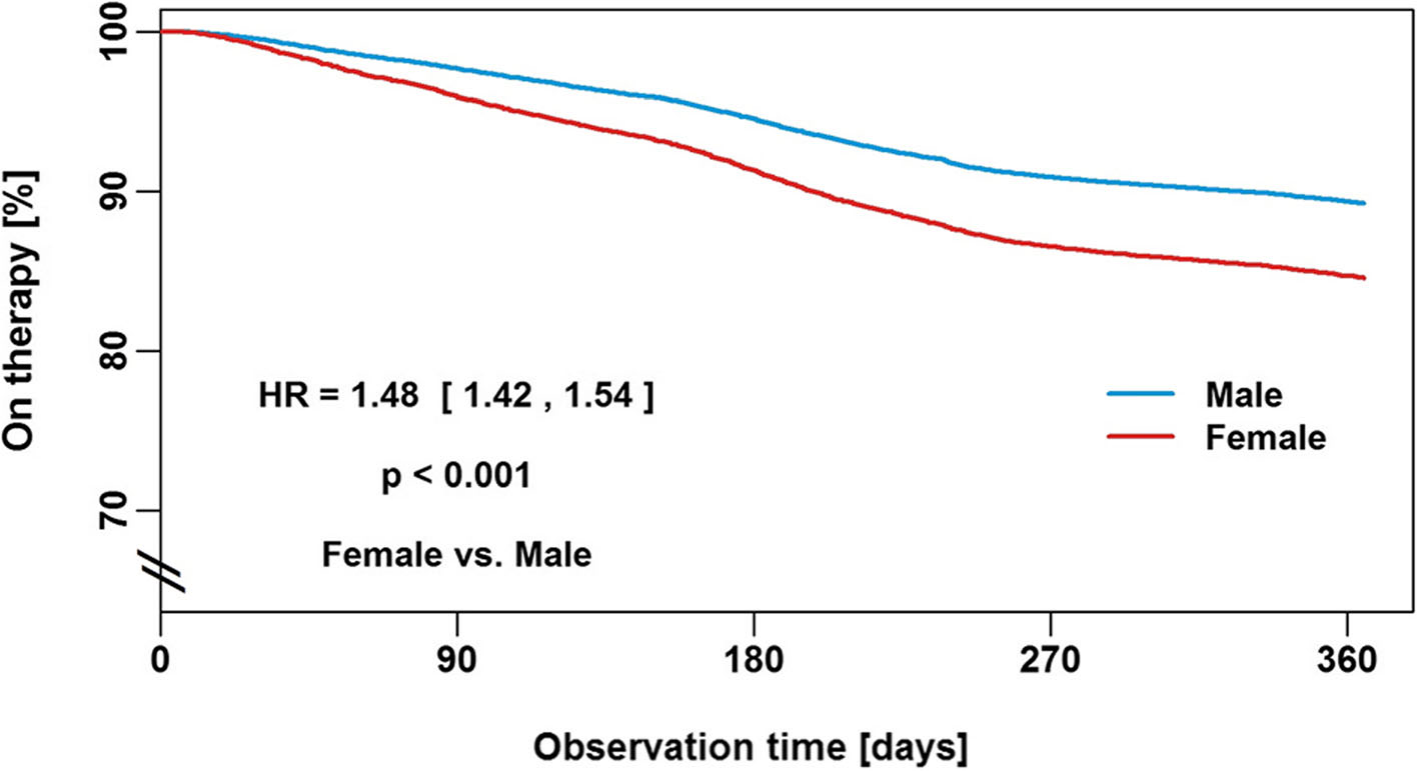
Time to therapy termination by gender (Cox proportional hazards regression). HR, hazard ratio

**Fig. 3:**
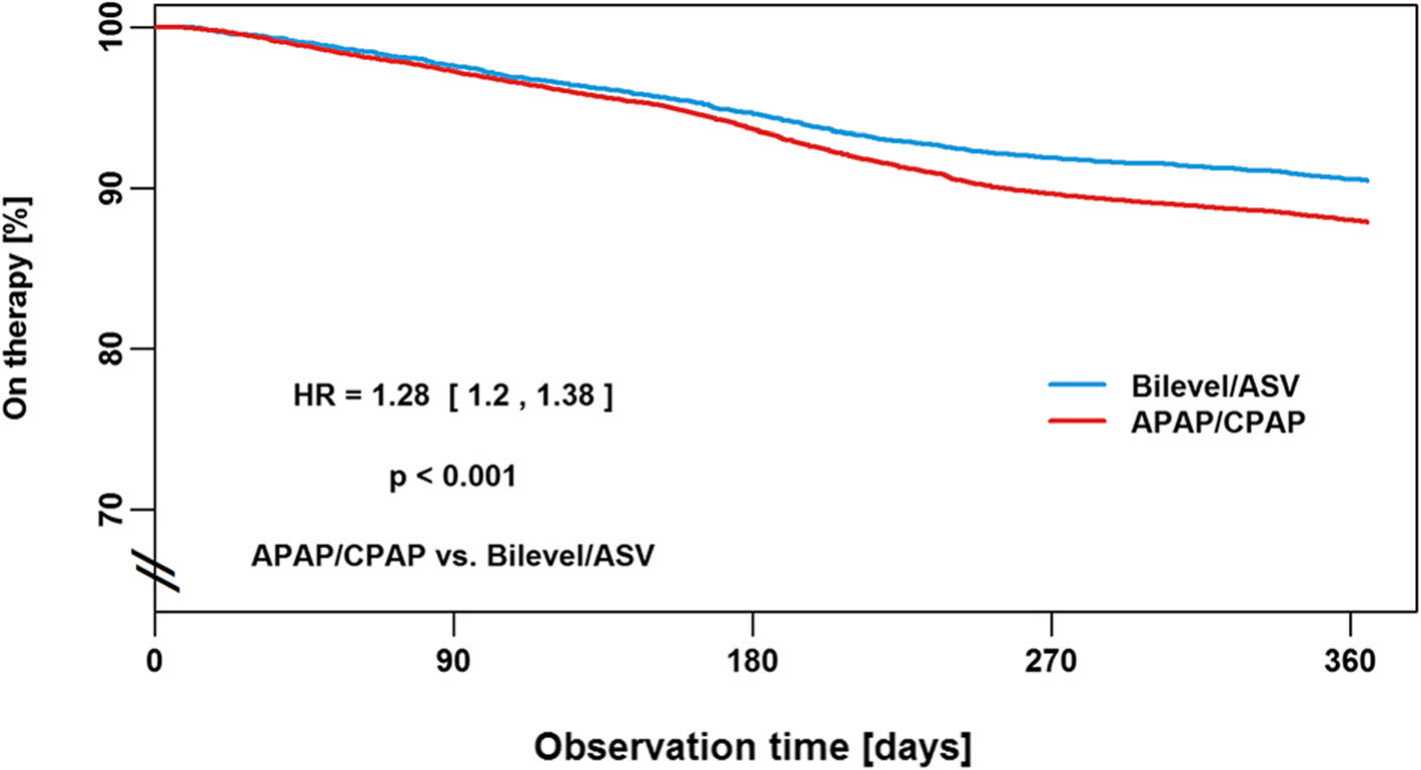
Time to therapy termination by first device used (Cox proportional hazards regression). APAP, automatic continuous positive airway pressure; ASV, adaptive servo-ventilation; Bilevel, bilevel positive airway pressure; CPAP, continuous positive airway pressure; HR, hazard ratio

**Fig. 4:**
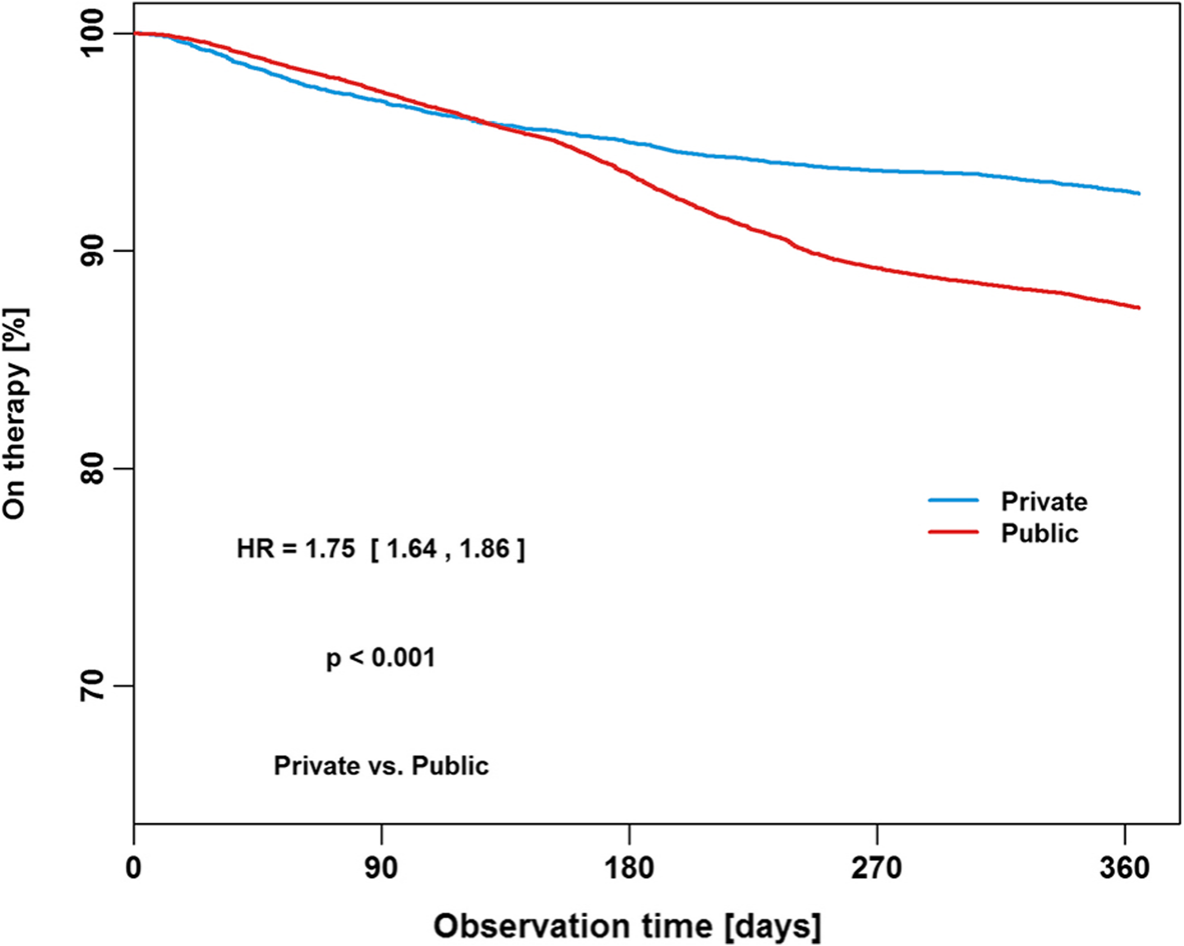
Time to therapy termination by insurance type (Cox proportional hazards regression). HR, hazard ratio

**Fig. 5:**
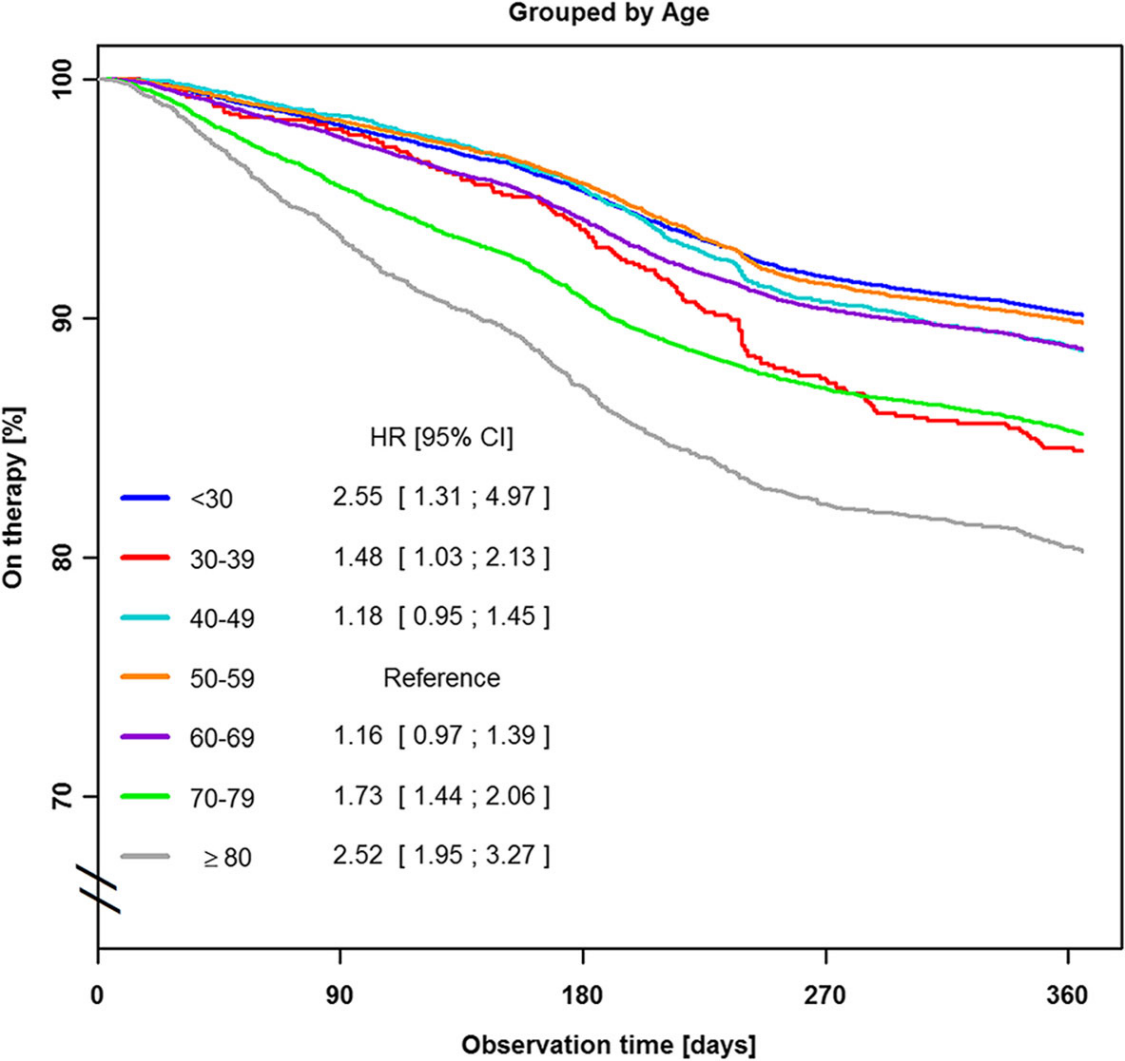
Time to therapy termination by patient age (Cox proportional hazards regression)

**Fig. 6:**
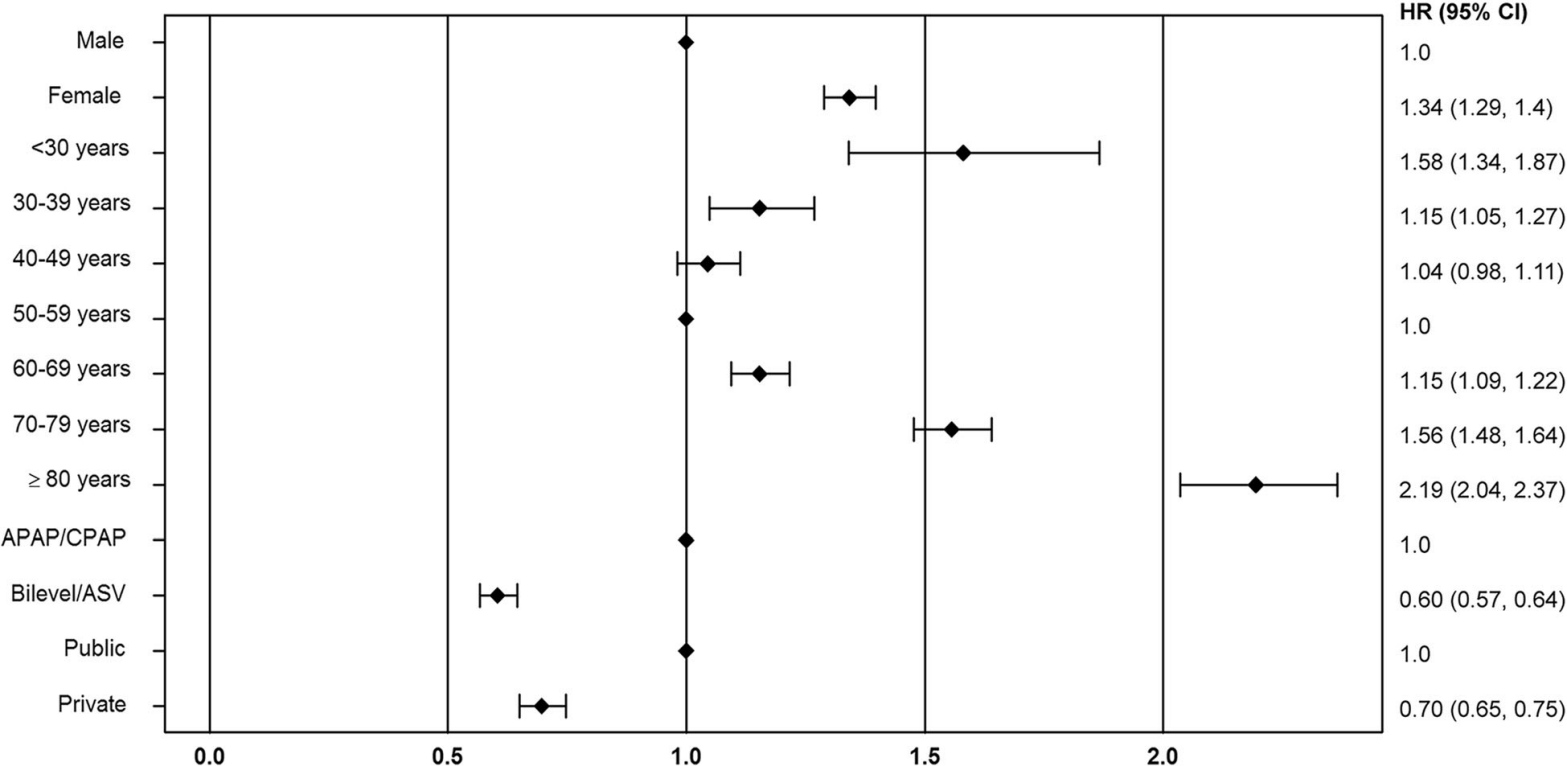
Forest plot of Cox model for time to therapy termination. APAP, automatic continuous positive airway pressure; ASV, adaptive servo-ventilation; Bilevel, bilevel positive airway pressure; CI, confidence interval; CPAP, continuous positive airway pressure; HR, hazard ratio

Reasons for therapy termination in the first year of PAP therapy were patient-related in 70% of subjects, administration- or insurance-related in 24%, based on medical decision in 1% and due to death in 5%. Cox proportional hazards regression analysis shows that the risk of therapy termination was significantly increased in female patients (hazard ratio [HR] 1.48, 95% confidence interval [CI] 1.42–1.54; *p* < 0.001; Fig. 2), when the first device was APAP or CPAP (HR 1.28, 95% CI 1.2–1.38; p < 0.001; Fig. 3), and when patients had public insurance (HR 1.75, 95% CI 1.64–1.86; Fig. 4). There was a U-shaped relationship between age and therapy termination rate. Compared with patients aged 50–59 years, the rate of therapy termination was significantly higher in younger patients (age < 30 years: HR 1.58, 95% CI 1.34–1.87; age 30–39 years: HR 1.15, 95% CI 1.05–1.27; *p* = 0.003) and in older patients (age 60–69 years: HR 1.15, 95% CI 1.10–1.22; p < 0.001; age 70–79 years: HR 1.56, 95% CI 1.48–1.64; p < 0.001; age ≥ 80 years: HR 2.19, 95% CI 2.04–2.37; p < 0.001); the rate of therapy termination in those aged 40–49 years did not differ significantly from that in those aged 50–59 years (HR 1.05, 95% CI 0.98–1.11; *p* = 0.167). Time to therapy termination in the different age groups is shown in Fig. 5. Presentation of the Cox model data in a Forest plot confirmed the above significant predictors of therapy termination, and highlights the U-shaped relationship between age and therapy termination (Fig. 6).

## Discussion

To the best of our knowledge, this big data analysis includes the largest dataset investigating predictors of current PAP therapy termination in practice to date. We identified a U-shaped relationship between age and therapy termination, with significantly higher therapy termination rates in younger and older age groups compared with patients aged 50–59 years. In geriatric patients aged 80 years or older, the therapy termination rate was double that in patients aged 50–59 years. Female patients were 1.4 times more likely to terminate therapy than males, while the risk of therapy termination was increased by 41% in patients who had public versus private insurance and there was a 26% higher rate of therapy termination in the first year when the first device was APAP or CPAP versus bilevel or ASV.

The overall PAP termination rate in the first year of therapy was 12% in our study, substantially lower than the 26% of patients who stopped PAP therapy in the first year of another large European analysis conducted in Switzerland (*n* = 2187) [29]. There are a number of factors that could have contributed to this difference, including the slightly newer technology used in our study, differences in diagnostic and treatment algorithms, and different patient population characteristics. In contrast to our findings and those of other studies [19, 30], age and gender were not significant independent predictors of PAP compliance in the Swiss study [29]. Instead, a low oxygen desaturation index (ODI) and Epworth Sleepiness Scale score, and high body mass index and apnea-hypopnea index were significantly associated with better compliance with PAP therapy [29]. Baseline sleep apnea severity has also been identified as a significant predictor of PAP compliance in a number of other studies [4, 19–23, 30], although the relationship has been described as relatively weak, especially when other factors are taken into account [28].

Existing data on the influence of age on compliance with CPAP therapy are conflicting. Increasing age has been shown to be associated with decreased CPAP usage [31]. However, this is far from a consistent observation, with several studies having failed to identify such an association [32–34], and older or increasing age has also been associated with better nightly CPAP usage [23, 35]. It has been suggested that other factors might attenuate the effects of advancing age on PAP compliance [22, 28]. The U-shaped relationship identified for the first time in our analysis could be one possible contributor to the inconsistent results reported to date. Only large data sets like ours allow five different age groups to be analyzed separately, which resulted in the identification of a U-shaped relationship between age and therapy termination. It would not be possible to identify such a relationship in studies comparing only very old and very young patients.

The results of the present analysis indicated a higher therapy termination rate in women compared with men. Evidence from existing literature in this area is again inconsistent. Although many studies have failed to find an association between gender and PAP usage [19, 25, 30, 36], others have shown female gender to be significantly associated with both better [23, 32] and worse [22, 37] CPAP compliance. The results of the current larger analysis identified female gender as a significant predictor of PAP therapy termination (i.e. poor compliance with therapy). Clearly the role of gender in compliance with PAP therapy is a topic that needs to be investigated further. Reasons underlying the higher therapy termination rate we observed in women versus men are also not clearly defined. It is possible that women dislike the aesthetics of PAP treatment more than men or have less tolerant partners. A dislike of the PAP therapy equipment could also be one potential explanation for higher therapy termination in the youngest group of patients. Furthermore, privately insured patients may have a better awareness of the cost of PAP therapy, increasing the likelihood of persevering with therapy. However, these suggestions are speculative and hypothesis generating only, and need to be investigated in future studies.

Interestingly, compliance rates in the long-term Swiss analysis that collected data over the period 2001 to 2011 were significantly higher in patients who started PAP therapy in the final 2 years of data analysis compared with those whose therapy was initiated earlier [29]. One possible explanation for this is that improvements in technology over time contributed to better compliance, highlighting the importance of data obtained in patients being treated with the latest devices and technologies.

This appears to be the first study to use big data to investigate predictors of PAP therapy termination. Analysis of big data allows inclusion of a very large population of patients and means that subgroup analyses include adequate numbers of patients to allow statistically meaningful comparisons. However, there are also some limitations associated with conducting scientific research using databases that were created for administrative, rather than scientific, purposes [26]. Such databases, including the one used in this analysis, include limited baseline clinical and demographic data, and no information about the severity of sleep-disordered breathing, method of diagnosis and comorbidities. The retrospective nature of the analysis is another limitation. In addition, compared with previous studies, we examined the rate of therapy termination rather than compliance. Therapy termination represents the most extreme form of non-compliance. There are other usage and behavior patterns that might fulfill criteria for non-compliance but not therapy termination, such as keeping the PAP device but never using it. The impact of telemonitoring on compliance with any type of PAP therapy was not evaluated in the current analysis (patients undergoing telemonitoring were excluded), but the impact of different telemedicine strategies on therapy termination rates have been reported separately [38, 39]. It is also important to note that the type of PAP device used depends on the type of sleep-disordered breathing being treated, with fixed pressure or automatically-titrating CPAP used to manage OSA, while bilevel PAP and ASV are better options when central sleep apnea is persistent or emerges during CPAP. Use of different devices in different settings could contribute to differences in compliance and therapy termination rates. Strengths of this analysis include the very large data set and the inclusion of a significant number of elderly and younger patients, and a large number of females. These groups are often under-represented in clinical trials, but were included in large numbers in this analysis, making the sample highly representative of clinical practice and improving the generalizability of the results. Nevertheless, caution needs to be exercised in extrapolating the results to other healthcare settings where differences in clinical practice might influence therapy termination rates [40]. Another important point to note is that our study analyzed different PAP devices together. The majority of previous studies investigating predictors of compliance have focused on CPAP, and variations in predictors of therapy termination between the different types of therapy (i.e. CPAP, ASV, etc.) cannot be ruled out. Nevertheless, the exploratory results are based on a very broad range of patients and provide useful information about which patient groups could be targeted to improve continuous use of PAP therapy, and serve as a guide for generating hypotheses and designing studies for future research.

## Conclusions

In this big database analysis of a large, unselected group of anonymized patients receiving PAP therapy in a real-world setting, gender, age, type of insurance, and device type were associated with therapy termination. These exploratory data highlight the importance of individualized approaches to PAP therapy management, and could help health care providers identify specific patient phenotypes that are at higher risk of stopping PAP therapy in the first year after treatment initiation. This would facilitate the choice of most appropriate PAP modality, targeting of interventions to support ongoing therapy to these groups, maximizing both the efficiency of resource use, service provision, and patient outcomes.

## Abbreviations

APAP: automatically adjusting continuous positive airway pressure
ASV: adaptive servo-ventilation
CI: confidence interval
CPAP: continuous positive airway pressure
HR: hazard ratio
OSA: obstructive sleep apnea
PAP: positive airway pressure
SD: standard deviation
